# Molecular Modeling Studies of Thiophenyl C-Aryl Glucoside SGLT2 Inhibitors as Potential Antidiabetic Agents

**DOI:** 10.1155/2014/739646

**Published:** 2014-12-10

**Authors:** Mukesh C. Sharma, Smita Sharma

**Affiliations:** ^1^School of Pharmacy, Devi Ahilya University, Takshila Campus, Khandwa Road, Indore 452 001, India; ^2^Department of Chemistry, Chodhary Dilip Singh Kanya Mahavidyalya, Bhind 477001, India

## Abstract

A QSAR study on thiophenyl derivatives as SGLT2 inhibitors as potential antidiabetic agents was performed with thirty-three compounds. Comparison of the obtained results indicated the superiority of the genetic algorithm over the simulated annealing and stepwise forward-backward variable method for feature selection. The best 2D QSAR model showed satisfactory statistical parameters for the data set (*r* ^2^ = 0.8499, *q* ^2^ = 0.8267, and pred_*r* ^2^ = 0.7729) with four descriptors describing the nature of substituent groups and the environment of the substitution site. Evaluation of the model implied that electron-rich substitution position improves the inhibitory activity. The good predictive 3D-QSAR models by *k-nearest neighbor (kNN)* method for molecular field analysis (MFA) have cross-validated coefficient *q* ^2^ value of 0.7663 and predicted *r* ^2^ value of 0.7386. The results have showed that thiophenyl groups are necessary for activity and halogen, bulky, and less bulky groups in thiophenyl nucleus enhanced the biological activity. These studies are promising for the development of novel SGLT2 inhibitor, which may have potent antidiabetic activity.

## 1. Introduction

One of the main features of diabetes is the elevation of blood sugar with its deleterious consequences in a variety of tissues [1]. Thus, control of the plasma glucose level is of utmost importance in the treatment of this disease. In recent years, the idea that affecting glucose absorption in the intestine and/or the glucose reabsorption in the kidney might be a possible way to control the sugar level has evolved. Diabetes comprises a group of metabolic disorders characterised by chronic hyperglycaemia with disorders in the metabolism of carbohydrate, fat, and protein that result in defects in secretion and action of insulin [2]. Dysfunction and failure of various organs, especially the eyes, kidneys, nerves, and heart, and the blood vessels are the usual complications of diabetes [3, 4]. Diabetes is mainly divided into four main types including insulin-dependent diabetes mellitus (type 1), non-insulin-dependent diabetes mellitus (type 2), gestational diabetes, and other specific types [5]. Diabetes mellitus type 2 (T2DM) accounts for almost 90% of diabetes cases, with the property of insulin resistance and beta-cell dysfunction that induces hyperglycemia [6]. Medical complications associated with T2DM include cardiovascular disease, stroke, nephropathy, retinopathy, renal failure, and amputations of the extremities [7]. In recent years, much attention has been given to sodium-dependent glucose cotransporters (SGLTs), mediators of reabsorption of glucose in the human body. Sodium-dependent glucose cotransporter 2 (SGLT2) is a high-capacity, low-affinity transporter expressed selectively in the S1 domain of the proximal tubule in the kidney and is responsible for 90% of renal glucose reuptake. Sodium dependent glucose cotransporter 1 (SGLT1), on the other hand, is a low-capacity, high-affinity transporter distributed in the kidney, gut, and other tissues, responsible for the remaining 10% of glucose reuptake [8]. Na^+^-glucose cotransporter (SGLT) is a membrane protein that plays an important role in the reabsorption of glucose in the kidneys. Sodium-dependent glucose cotransporters (SGLTs), mediators of reabsorption of glucose in the kidney, have recently emerged as novel drug targets for the treatment of diabetes [9]. SGLT is known to have three isoforms (SGLT1, SGLT2, and SGLT3) [10–12]. SGLT1 is expressed primarily in the brush border membrane of mature enterocytes in the small intestine, where it absorbs dietary glucose and galactose from the gut lumen [13]. SGLT2, expressed exclusively in the kidney, is located in the S1 segment of the proximal convoluted tubule of the kidney. It is a low-affinity, high-capacity cotransporter and is responsible for 90% of renal glucose reabsorption [14]. Several therapeutic agents are available for monotherapy or for combination therapy with different mechanisms to treat diabetics, such as metformin, rosiglitazone, sitagliptin, acarbose, and glimepiride [15]. The obvious need for new approaches to treat patients with uncontrolled T2DM has prompted continuous exploration of alternative targets involved in maintenance of glucose homeostasis. Several SGLT2 inhibitors have been reported as undergoing clinical trials. Phlorizin [16], 3-(benzo[b]furan-5-yl)-2′,6′-dihydroxy-4′-methylpropiophenone-2′-O-*β*-D-glucopyranoside [17], sergliflozin [18], and remogliflozin [19] are O-glycosides and show strong inhibition of SGLT2. They also demonstrate efficacy* in vivo* when administered orally in rats or mice. They induce a glucosuric response, the result of the blockade of renal glucose reabsorption, and consequently lead to reduction of the blood glucose level and improvement of insulin sensitivity [20]. Quantitative structure-activity relationship (QSAR) studies can be utilized to predict eye irritation potential as an alternative* in silico* method, just as it has been used successfully to predict several other toxicological endpoints for some time [21]. Hence, in continuation to our efforts [22–67] in developing QSAR studies for angiotensin II AT_1_ receptor, antitubercular agents, antimalarial activity, antimicrobial activity, antibacterial activity, COX inhibitors, and so forth.

In this study, we have taken thiophenyl derivatives for performing 2D and 3D quantitative structural-activity relationship analysis and calculations in order to understand their stereoelectronic properties. Genetic algorithm (GA), simulated annealing (SA), and stepwise forward-backward variable selection methods have been employed for selection of relevant descriptors. The obtained results provide further insight into some beneficial information in structural modifications to design new potential SGLT2 inhibitors. Moreover, new compounds with high predictive activities were designed.

## 2. Materials and Methods

### 2.1. Data Set

The biological data set was chosen from a series of thirty-three thiophenyl derivatives as SGLT2 inhibitors as potential antidiabetic agents reported by Lee et al. [68]. The biological activity values [IC_50_ (nM)] reported in nanomolar units were converted to their molar units pIC_50_ and subsequently used as the dependent variable for the QSAR analysis. The converted to pIC_50_ for the QSAR analysis along with the structure of the compounds in the series are listed in Table 1 (marked with asterisk). The test compounds were selected manually such that the structural diversity and wide range of activity in the data set were included. In this paper, a series of thiophenyl compounds with substitutions at X and R position of thiophenyl moiety are subjected to examining the relationships between structural modifications and activities against hSGLT2 inhibitors with the help of QSAR modeling.

### 2.2. Computational Details

All the computational studies were performed by V-life MDS (Molecular Design Suite) 3.5 software supplied by V-life Sciences Technologies Pvt. Ltd., Pune, India [69]. The sketched structures were used for the calculation of 2D molecular descriptors using QSAR module of Molecular Design Suite software. Each compound was subjected to energy minimization and batch optimization using Merck Molecular Force Field (MMFF), fixing Root Mean Square Gradients (RMS) to 0.01 kcal/mol Å [70].

The sphere exclusion method [71] was adopted total set of inhibitors was divided randomly into a training set (26 compounds) for generation of QSAR models and a test set (7 compounds) for validation of the developed model. This random division of data set will be done through several cycles in order to get the best QSAR model. This study will help in rational drug designing of these derivatives as SGLT2 inhibitors for the eradication of T2DM. The unicolumn statistics of the training and test sets are reported in Table 2. The maximum and minimum value in training and test set were compared in a way that

1. the maximum value of pIC_50_ of test set should be less than or equal to maximum value of pIC_50_ of training set,
2. the minimum value of pIC_50_ of test set should be higher than or equal to minimum value of pIC_50_ of training set.

### 2.3. Calculating 2D Descriptors

In the current approach, an attempt has been taken to understand the structural and physicochemical requirements of a set of hSGLT2 inhibitors by the help of regression 2D quantitative structure-activity relationship (2D QSAR). The energy-minimized geometry was used for the calculation of the various 2D descriptors such as topological, shape and geometrical, and physicochemical parameters such as individual (H-Acceptor count, H-Donor count, XlogP, retention index (Chi), element count, estate numbers, estate contribution, and alignment-independent descriptors were used as predictor variables), as they were found to be appropriate for the development of models. A considerable number of the 265 physicochemical parameters, 300 alignment type parameters, and 99 atoms types count descriptors calculations were done using the V-life, MDS 3.5. The preprocessing of the independent variables (i.e., 2D descriptors) was done by removing the invariable (constant column) which resulted in a total of 216 molecular descriptors to be used for QSAR analysis. The various alignment-independent descriptors were also calculated. In this study to calculate AI descriptors, we have used the following attributes: 2 (double bonded atom), 3 (triple bonded atom), C, N, O, S, H, F, Cl, Br, and I, and the distance range of 0–7. The QSAR models were built with the consideration of the applicability of the descriptor for the activity. Various types of physicochemical descriptors have been calculated which are shown in the data sheet (Table 3).

### 2.4. Calculating 3D Descriptors

**Figure 1:**
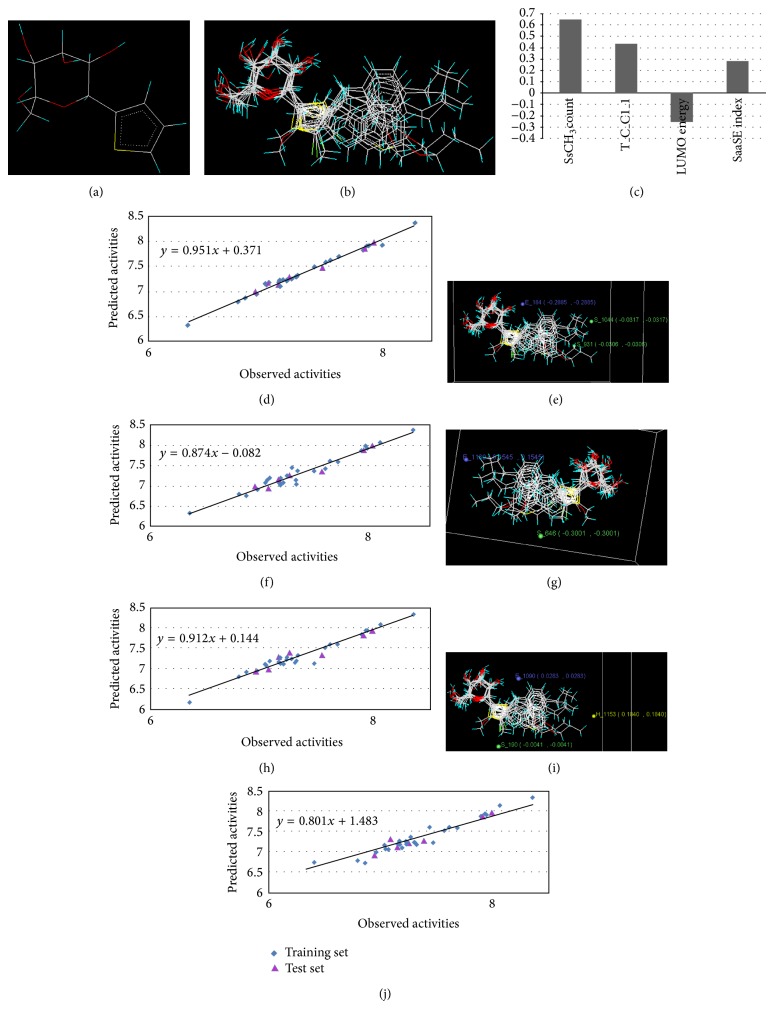
(a) Thiophenyl ring (template structure). (b) Alignment of thiophenyl derivatives. (c) Contribution charts of the descriptors for the 2D QSAR model-1. (d) Plot of observed* versus* predicted activity by 2D QSAR model-1. (e) Contribution plot for steric and electrostatic interactions GA-PLS model. (f) Plot of observed* versus* predicted activity by 3D QSAR GA-PLS model. (g) Contribution plot for steric and electrostatic interactions SA-PLS model. (h) Plot of observed* versus* predicted activity by 3D QSAR SA-PLS model. (i) Contribution plot for steric, hydrophobic, and electrostatic interactions SW-PLS model. (j) Plot of observed* versus* predicted activity by 3D QSAR SW-PLS model.

Energy minimized and geometry optimized structures of molecules were aligned by the template-based method [72] where a template structure is defined and used as a basis for alignment of a set of molecules, and a reference molecule is chosen on which the other molecules of the data set get aligned considering the chosen template. In the present study, we aligned the database by fitting all of the compounds on most active compound 16 (Figure 1(a)) as an alignment template using a common substructure with the V-life MDS routine database alignment. The superimposition of all molecules based on minimizing root mean square deviation (RMSD) is shown in Figure 1(b).

The steric, electrostatic, and hydrophobic fields were calculated at each lattice intersection of a regularly spaced grid of 2.0 Å. Methyl probe of charge +1 with 10.0 kcal/mole electrostatic and 30.0 kcal/mole steric and hydrophobic cutoff was used for fields generation. This resulted in calculation of 4500 field descriptors (1500 for each steric, electrostatic, and hydrophobic which theoretically form a continuum) for all the compounds in separate columns (Table 3).

### 2.5. External Validation for 2D QSAR Models

The QSAR models were assessed by the number of cross-validated *R* ^2^ by leave-one-out method [73] (*q* ^2^), cross-validated standard error (*q* ^2^_se), predicted *R* ^2^ for external test set (pred_*r* ^2^), and standard error for predicted *R* ^2^ (pred_*r* ^2^se). The internal cross-validated predictability (*q* ^2^) was evaluated by the equation given below:

(1) $$\begin{array}{c} q^{2}=1-\frac{\sum {\left(y_{i\;}-{\hat{y}}_{i}\right)}^{2}}{\sum {\left(y_{i}-y_{\text{mean}}\right)}^{2}}, \end{array}$$

where $y_{i},\hat{y_{i}}$ are the actual and predicted activity of the *i*th molecule in the training set, respectively, and *y*_mean_ is the average activity of all molecules in the training set. For external validation, activity of each molecule in the test set was predicted using the model generated from the training set. The pred_*r* ^2^ value is calculated as follows:

(2) $$\begin{array}{c} \text{Pred}\_r^{2}=1-\;\frac{\sum {\left(y_{i\;}-{\hat{y}}_{i}\right)}^{2}}{\sum {\left(y_{i}-y_{\text{mean}}\right)}^{2}}, \end{array}$$

where $y_{i},\hat{y_{i}}$ are the actual and predicted activity of the *i*th molecule in the test set, respectively, and *y*_mean_ is the average activity of all molecules in the training set. Both summations are over all molecules in the test set. Thus, the pred_*r* ^2^ value is indicative of the predictive power of the current model based on the external test set.

### 2.6. Evaluation of the Quantitative of Models

Among several search algorithms, stepwise (SW) forward variable selection method, genetic algorithms (GA), and simulated annealing (SA) based feature selection procedures are most popular for building QSAR models and can explain the situation more effectively. The models were also subjected to the test for criteria of external validation as suggested by Golbraikh and Tropsha [71]. To know predictive potential of the models, squared correlation coefficient values between the observed and predicted values of the test set compounds with intercept (*r* ^2^) were calculated. Interchange of the axes gives the value of *r* ^2^. According to Golbraikh and Tropsha, models are considered acceptable, if they satisfy all of the following conditions:

1. *Q* ^2^ > 0.5,
2. *r* ^2^ > 0.6.

When the observed values of the test set compounds (*y*-axis) are plotted against the predicted values of the compounds (*x*-axis) setting intercept to zero, the slope of the fitted line gives the value of *k*.

## 3. Results and Discussion

QSAR study was performed on thiophenyl C-aryl glucoside derivatives for their SGLT2 inhibitors as potential antidiabetic agents. Comparison of the obtained results indicated the superiority of the genetic algorithm over the simulated annealing (SA) and stepwise forward-backward variable method for feature selection:

 pIC_50_ = 0.6451 (±0.1584) SsCH_3_count + 0.4287 (±0.0851) T_C_Cl_1 − 0.2574 (±0.0036) LUMO energy + 0.2789 (±0.0487) SaaSE-index;
 
*N*_training_ = 26, *N*_test_ = 7, degree of freedom = 21, *r* ^2^ = 0.8499, *q* ^2^ = 0.8267, *F*-test = 45.8975, *r* ^2^_se = 0.3098, *q* ^2^_se = 0.3464, pred_*r* ^2^ = 0.7729, and pred_*r* ^2^se = 0.6158.

The significant model with *r* ^2^ = 0.8499 was considered, as model-1 showed an internal predictive power (*q* ^2^ = 0.8267) and a predictivity for the external test set (pred_*r* ^2^ = 0.7729) of about 77%. The *F*-test = 45.897 shows the statistical significance of 99.99% of the model which means that the probability of failure of the model is 1 in 10000. In addition, the randomization test shows confidence of ~99.9% that the generated model is not random and hence it is chosen as the QSAR model. Genetic algorithm-PLS model indicates the positive contribution of SsCH_3_count, and SaaSE-index showed that increase in the values of these descriptors is beneficial for the SGLT2 inhibitors. From the above model, it is clear that the descriptor T_C_Cl_1 contributes positively to the SGLT2 inhibitors activity, which corresponds to count of number of carbon atoms separated from any chlorine atom by 1-bond distance. Thus, the presence of chloro substituents (like in compounds 1–18) would increase the activity. Descriptor SsCH_3_count indicates that increase in methyl group of R position may lead to an increase in the activity. Its positive value suggests that increasing the number of such carbons will lead to better SGLT2 inhibitors. This suggests that substituents such as methyl would increase the activity. The above results are in close agreement with the experimental observations, where compounds 10, 15, 16, 17, and 28–33 at the R position produce SGLT2 inhibitors. Molecules with negative coefficient LUMO energy can accept electrons more easily than those having high LUMO energy. The SaaSE-index (~24%) shows the sulphur atom connected with two aromatic bonds in the molecule and is inversely proportional to the activity. This further suggests that the increase in electronegative atom environment adjacent to indicated sulphur atom would result in increase in the activity. In addition, in agreement with QSAR model, presence of more bulky and hydrophilic substituents like methoxy at ring R led to an increase in SGLT2 potency. On the other hand, more lipophilic halogens like chloro in this X position retain the SGLT2 inhibition activities. The residuals (observed-predicted activity) were found to be minimal and are presented in Table 4. The statistical results of best model and the correlation matrix between the physicochemical parameters and the biological activity for model-1 are presented in Table 5. The contribution chart of selected descriptors is represented in Figure 1(c). Also, the graph for observed* versus* predicted activity for the series is plotted in Figure 1(d) which shows good correlation.

Molecular fields are the steric, electrostatic, and hydrophobic interaction energies which are used to develop a model for 3D QSAR. In this study, 3D QSAR models were generated by kNN-MFA in conjunction with genetic algorithms (GA), simulated annealing (SA), and stepwise (SW) forward-backward selection methods:

 pIC_50_ = S_1044 (−0.0317, −0.0317) − E_184 (−0.2885, −0.2885) − S_931 (−0.0306, −0.0306);
 
*N*_training_ = 26, *N*_test_ = 7, *q* ^2^ = 0.7663, *q* ^2^_se = 0.3389, *F*-test = 38.4683, and pred_*r* ^2^ = 0.7386.

The steric and electrostatic interaction energies are computed at the lattice points of the grid using a methyl probe of charge +1. The best GA-kNN MFA 3D QSAR model that has a *q* ^2^ of 0.7663 and pred_*r* ^2^ of 0.7386 was considered. The points generated in GA-kNN MFA 3D QSAR model are S_1044 (−0.0317, −0.0317), E_184 (−0.2885, −0.2885), and S_931 (−0.0306, −0.0306).  Figure 1(e) shows the contribution plot of the three models for the electrostatic and steric fields, respectively, and indicates relative regions of the local fields around the aligned molecules leading to activity variation in the model. For electrostatic field and steric fields, the lattice points generated in the model are E_184 (−0.2885, −0.2885) and S_1044 (−0.0317, −0.0317) and S_931 (−0.0306, −0.0306). These points suggested the significance of electrostatic properties as indicated in the ranges in parentheses for maximum SGLT2 inhibitory activity. The negative value for E_184 means that electron-withdrawing substituents in this region are favorable and would increase SGLT2 inhibitory activity, as shown by the presence of chlorine group in the active compounds. Therefore, less steric and more steric substituents were preferred at the position of generated data points S_1044 and S_931, respectively, for enhancing the biological activity of thiophenyl pharmacophore. Two data points generated at the position of R around thiophenyl nucleus were steric points S 1044 and S 931 which indicates that less steric or less bulky substituents are favorable on this site. On the other hand, less electronegative groups such as hydroxyl and nitro. The electrostatic blue ball model around R positions of the thiophenyl suggested the electron-withdrawing groups on this position benefited potency; this may be the reason why compounds with double bonds at R positions had higher potencies than other compounds. In addition, a red contour near the position suggested the electron-withdrawing substituent would increase the activity. Therefore, the –OH at R position resulted in significant increased activity. Electron-withdrawing nature of the electronegative chloro atom does contribute to the SGLT2 inhibitory activity of the molecule. The graph for observed* versus* predicted activity for the series is plotted in Figure 1(f) which shows good correlation. The residuals (observed-predicted activity) were found to be minimal and are presented in Table 4:

 
*N*_training_ = 26, *N*_test_ = 7, *q* ^2^ = 0.7254, *q* ^2^_se = 0.3795, *F*-test = 31.8965, and pred_*r* ^2^ = 0.6938.

3D data points generated, which contribute to SA kNN-MFA QSAR model, are shown in Figure 1(g). The external predictability of the above 3D QSAR model using the test set was determined by Pred_*r* ^2^, which is 0.6938. The points generated in SA kNN-MFA 3D QSAR model are E_1139 (0.1545, 0.1545) and S_646 (−0.3001, −0.3001), that is, electrostatic and steric interaction fields at lattice points 1139 and S_646, respectively. Positive values for E_1139 show that electron-donating groups on the thiophenyl ring increase biological activity of compounds. On the other hand, thiophenyl ring might be substituted with either electron-withdrawing or electron-donating groups without loss of activity. 3D QSAR studies showed requirement of steric group at R position. The graph for observed* versus* predicted activity for the series is plotted in Figure 1(h). The residuals (observed-predicted activity) were found to be minimal and are presented in Table 4:

 
*N*_training_ = 26, *N*_test_ = 7, *q* ^2^ = 0.7359, *q* ^2^_se = 0.3562, *F*-test = 30.8743, and pred_*r* ^2^ = 0.6743.

The 3D model using the SW-kNN-MFA analysis method *q* ^2^ was found to be 0.7359 which suggests that the model could be useful for predicting SGLT2 inhibitor for such thiophenyl derivatives. These points suggested the significance and requirement of electrostatic and steric and hydrophobic properties as mentioned in the ranges for structure-activity relationship and biological activity of thiophenyl analogues. It shows a positive contribution towards the activity, which indicates that H_1153 hydrophobic substitution at R position favors activity.  Figure 1(i) showed the hydrophobic contour the presence of a yellow contour covering the R positions of the ring indicating that hydrophobic substituents may be well tolerated in that region. The R position of the thiophenyl was surrounded by a yellow contour which also indicated that hydrophobic groups at this position may increase activity. Less bulky substituents are tolerated at thiophenyl ring meaning that increasing size of the groups substituted in these regions reduces SGLT2 inhibitory activity, since S_190 have negative values. Positive values for the hydrophobic 1153 lattice points around ring indicate that SGLT2 inhibitory activity could be increased by substituting more hydrophobic groups in these regions. Similarly, the positive values of electrostatic descriptors suggested the requirement of electropositive or sterically bulky groups at the position of generated data point E_1090 around thiophenyl pharmacophore for maximum activity. The electrostatic E_1090 data point generated which indicates sterically bulky groups such as benzene, methyl, ethyl, and isopropyl was required at R position. The graph for observed* versus* predicted activity for the series is plotted in Figure 1(j). The residuals (observed-predicted activity) were found to be minimal and are presented in Table 4.

## 4. Conclusions 

QSAR study was performed on thiophenyl C-aryl glucoside derivatives for their SGLT2 inhibitors as potential antidiabetic agents. Genetic algorithms (GA), simulated annealing (SA), and stepwise (SW) forward-backward selection methods have been employed for selection of relevant descriptors. Comparison of the obtained results indicated the superiority of the genetic algorithm over the stepwise method for feature selection. 2D QSAR further revealed that a specific group or type of descriptor is not sufficient to capture the true factors responsible for the activity in the set of inhibitor compounds. This study also revealed that SsCH_3_count, along with LUMO energy and SaaSE-index, forms a powerful tool to improve a QSAR model. This study used T_C_Cl_1 to investigate whether a similarity based set generation method would lead to better understanding of the QSAR models. The 2D and 3D QSAR suggested the presence of negative steric potential at R position in thiophenyl nucleus, that is, R position in thiophenyl nucleus should acquire less steric or less bulky substituents are favorable as well as according to models. The constructed 3D QSAR models and structure-activity relationship (SAR) analyses of the compounds used in the study suggested that an electronegative and bulky substituent at R position and one less bulky substituent at X, R position of thiophenyl analogs are required to design novel SGLT2 inhibitors. We emphasize in this study that the E_1139 electron-donating groups on the thiophenyl ring increase biological activity of compounds. Electron-withdrawing groups are highly favorable. Furthermore, the kNN-MFA maps offered enough information to understand the structure-activity relationship and identified structural features influencing the inhibitory activity. The correlation of the results obtained from 3D QSAR study successfully explored the primitive structure-activity relationship. The findings can be quite useful to aid the designing of novel antidiabetic agents with high predicted potent activity.

## Figures and Tables

**Table 1:** Structure and biological activity of thiophenyl derivatives hSGLT2 inhibitors.

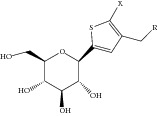				
S. number	X	R	IC_50_	pIC_50_
1	Cl	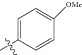	86.5	7.0629
2	Cl	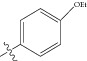	34.6	7.4609
3^*^	Cl	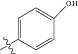	140	6.8538
4	Cl	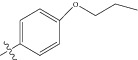	65.0	7.1870
5	Cl	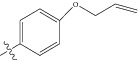	54.6	7.2628
6^*^	Cl	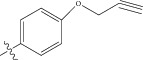	111	6.9546
7	Cl	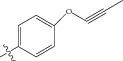	94	7.0268
8	Cl	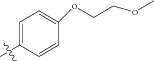	115	6.9393
9	Cl	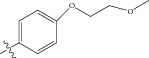	70.7	7.1505
10^*^	Cl	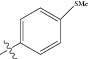	12.8	7.8927
11	Cl	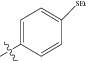	48.4	7.3151
12	Cl	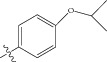	11.9	7.9244
13	Cl	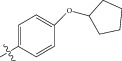	57.2	7.2426
14^*^	Cl	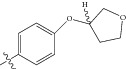	162	6.7904
15	Cl	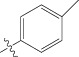	60.2	7.2204
16	Cl	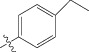	4.47	8.3496
17	Cl	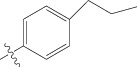	10.3	7.9871
18	Cl	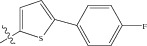	91.3	7.0395
19^*^	H	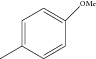	11.5	7.9393
20	H	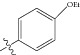	8.73	8.0589
21	H	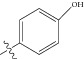	50.2	7.2992
22^*^	H	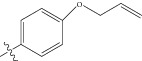	27.5	7.5606
23	H	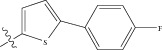	21.1	7.6757
24	H	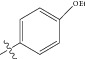	71.4	7.1463
25^*^	H	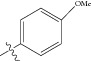	68.9	7.1617
26	H	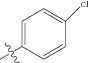	451	6.3458
27	H	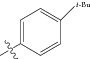	88.3	7.0540
28	H	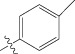	69.6	7.1573
29^*^	H	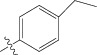	24.8	7.6055
30	H	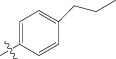	59.8	7.2232
31	Br	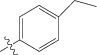	12.4	7.9065
32	OMe	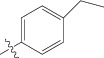	49.5	7.3053
33^*^	Me	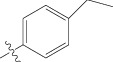	29.3	7.5331

^*^Structures with (^*^) are included in test set validation.

**Table 2:** Unicolumn statistics of training and test set for 2D QSAR studies.

Data set	Average	Max	Min	Std. dev.	Sum
2D QSAR					
Training	1.6127	2.6542	0.6503	0.4426	41.9299
Test	1.7765	2.2095	1.1072	0.3889	12.4355

3D QSAR					
Training	1.5401	2.2095	0.6503	0.4163	36.9636
Test	1.9335	2.6542	1.3945	0.3452	17.4018

**Table 3:** Selected descriptors values for QSAR models.

SsCH_3_count	T_C_Cl_1	S_1044	E_184	E_1139	H_1153
13.35725	1	−0.00234	−0.00103	0.070427	0.068996
14.89175	1	−0.00252	−0.03374	0.039663	0.110293
13.05024	1	−0.00332	−0.0034	0.045418	0.156499
12.66982	1	−0.00271	−0.07924	0.015991	0.205079
13.19176	1	−0.00718	−0.05341	0.01499	0.156726
13.4038	1	−0.00276	−0.02181	0.126288	0.213233
13.05024	1	−0.00272	−0.11568	0.049628	0.170716
14.11489	1	−0.00229	−0.04659	0.03041	0.190254
13.19176	1	−0.0037	−0.05161	0.007637	0.195659
13.85076	1	−0.00269	−0.09992	0.148325	0.170358
13.95209	1	−0.002	−0.05398	0.046206	0.178527
13.49164	1	−0.00307	−0.04381	0.022364	0.196138
13.8452	1	−0.00213	−0.03588	0.01794	0.135694
13.11122	1	−0.00219	−0.05675	0.025435	0.117383
11.57052	1	−0.0077	−0.03111	0.040108	0.13964
13.05024	1	−0.00148	−0.06435	0.079842	0.242054
13.05024	1	−0.00131	−0.03439	0.040536	0.219392
12.37104	1	−0.00205	−0.00201	0.013029	0.200942
13.85076	0	−0.00288	−0.02182	0.060327	0.072212
13.49164	0	−0.00392	−0.07865	0.052315	0.116761
11.65703	0	−0.00194	−0.0367	0.068249	0.151754
12.45755	1	−0.00638	−0.03708	0.025638	0.202708
13.35725	0	−0.00325	−0.0005	0.016382	0.181212
12.3107	0	−0.00272	−0.06519	0.139852	0.220012
13.11122	0	−0.00186	−0.04364	0.073036	0.168351
12.30098	0	−0.00406	−0.01576	0.034676	0.18917
13.1015	1	−0.00226	−0.03521	0.000739	0.211024
11.56143	1	−0.00212	−0.07403	0.159932	0.171132
12.11965	1	−0.00331	−0.02547	0.047064	0.188637
12.3107	1	−0.00178	−0.03979	0.004465	0.186037
12.92017	1	−0.0024	−0.03588	0.030881	0.137912
13.11122	1	−0.01145	−0.05675	0.032062	0.126376
13.35725	1	−0.00109	−0.00103	0.070427	0.137455
14.89175	1	−0.0011	−0.03374	0.039663	0.232216
13.05024	1	−0.00082	−0.0034	0.045418	0.257901
12.66982	1	−0.00144	−0.07924	0.015991	0.22212
13.19176	1	−0.00178	−0.05341	0.01499	0.144703
13.4038	1	−0.00071	−0.02181	0.126288	0.132103
13.05024	1	−0.00234	−0.11568	0.049628	0.068996
14.11489	1	−0.00252	−0.04659	0.03041	0.110293
13.19176	1	−0.00332	−0.05161	0.007637	0.156499

**Table 4:** Comparative observed and predicted activities (LOO) of thiophenyl SGLT2 inhibitors.

Com	pIC_50_	2D model-1		3D model-GA-PLS		3D model-SA-PLS		3D model-SW-PLS	
		Pred.	Res.	Pred.	Res.	Pred.	Res.	Pred.	Res.
1	7.0629	7.1845	−0.1216	7.1847	−0.1218	7.1718	−0.1089	7.0553	0.0076
2	7.4609	7.4978	−0.0369	7.3679	0.093	7.1283	0.3326	7.2289	0.232
3	6.8538	6.8737	−0.0199	6.7565	0.0973	6.9154	−0.0616	6.7295	0.1243
4	7.187	7.2319	−0.0449	7.0789	0.1081	7.1122	0.0748	7.1047	0.0823
5	7.2628	7.2564	0.0064	7.4543	−0.1915	7.2428	0.02	7.3525	−0.0897
6	6.9546	6.9448	0.0098	6.9121	0.0425	6.9393	0.0153	6.9875	−0.0329
7	7.0268	7.1464	−0.1196	7.0723	−0.0455	7.1174	−0.0906	7.1653	−0.1385
8	6.9393	6.9815	−0.0422	6.9644	−0.0251	6.9163	0.023	6.9128	0.0265
9	7.1505	7.1854	−0.0349	7.0746	0.0759	7.1649	−0.0144	7.1761	−0.0256
10	7.8927	7.8289	0.0638	7.8694	0.0233	7.8265	0.0662	7.8656	0.0271
11	7.3151	7.3283	−0.0132	7.3729	−0.0578	7.3247	−0.0096	7.1826	0.1325
12	7.9244	7.9135	0.0109	7.9918	−0.0674	7.9418	−0.0174	7.9356	−0.0112
13	7.2426	7.2857	−0.0431	7.2498	−0.0072	7.3821	−0.1395	7.2148	0.0278
14	6.7904	6.7845	0.0059	6.7954	−0.005	6.7991	−0.0087	6.7894	0.001
15	7.2204	7.2138	0.0066	7.2525	−0.0321	7.2862	−0.0658	7.1966	0.0238
16	8.3496	8.3791	−0.0295	8.3875	−0.0379	8.3424	0.0072	8.3446	0.005
17	7.9871	7.9845	0.0026	7.9949	−0.0078	7.9326	0.0545	7.9674	0.0197
18	7.0395	7.1278	−0.0883	7.1392	−0.0997	7.0734	−0.0339	7.0673	−0.0278
19	7.9393	7.9243	0.015	7.9418	−0.0025	7.9388	0.0005	7.9132	0.0261
20	8.0589	7.9275	0.1314	8.0692	−0.0103	8.0872	−0.0283	8.1471	−0.0882
21	7.2992	7.2961	0.0031	7.1491	0.1501	7.1449	0.1543	7.2307	0.0685
22	7.5606	7.5884	−0.0278	7.4281	0.1325	7.5161	0.0445	7.5319	0.0287
23	7.6757	7.6978	−0.0221	7.5946	0.0811	7.5988	0.0769	7.5975	0.0782
24	7.1463	7.1326	0.0137	7.1384	0.0079	7.2864	−0.1401	7.1125	0.0338
25	7.1617	7.0968	0.0649	7.1948	−0.0331	7.1289	0.0328	7.1799	−0.0182
26	6.3458	6.3291	0.0167	6.3259	0.0199	6.1643	0.1815	7.4332	−0.0874
27	7.054	7.1853	−0.1313	6.9342	0.1198	6.9768	0.0772	6.9087	0.1453
28	7.1573	7.2218	−0.0645	7.0202	0.1371	7.2738	−0.1165	7.2684	−0.1111
29	7.6055	7.6283	−0.0228	7.6163	−0.0108	7.6023	0.0032	7.6132	−0.0077
30	7.2232	7.2346	−0.0114	7.2458	−0.0226	7.2282	−0.005	7.2547	−0.0315
31	7.9065	7.8579	0.0486	7.8881	0.0184	7.8194	0.0871	7.8658	0.0407
32	7.3053	7.2982	0.0071	7.0424	0.2629	7.1921	0.1132	7.8928	−0.5875
33	7.5331	7.4728	0.0603	7.3466	0.1865	7.3308	0.2023	7.2733	0.2598

**Table 5:** Correlation matrix between descriptors present in the best QSAR model-1.

Parameter	pIC_50_	SsCH_3_count	T_C_Cl_1	LUMO energy	SaaSE-index
pIC_50_	1.0000				
SsCH_3_count	0.5431	1.0000			
T_C_Cl_1	0.2165	0.4312	1.0000		
LUMO energy	0.4964	0.5731	0.6823	1.0000	
SaaSE-index	0.3731	0.4633	0.6591	0.8504	1.0000
